# A global set of Fourier-transformed remotely sensed covariates for the description of abiotic niche in epidemiological studies of tick vector species

**DOI:** 10.1186/1756-3305-7-302

**Published:** 2014-07-02

**Authors:** Agustín Estrada-Peña, Adrián Estrada-Sánchez, José de la Fuente

**Affiliations:** 1 Dept. of Animal Pathology, University of Zaragoza, Miguel Servet 177, Zaragoza 50013, Spain; 2 Department of Geograohy, University of Zaragoza, Zaragoza Spain; 3 SaBio, IREC-CSIC-UCLM-JCCM, University of Castilla-La Mancha, Ciudad Real, Spain; 4 Department of Veterinary Pathobiology, Center for Veterinary Health Sciences, Oklahoma State University, Stillwater, OK 74078, USA

**Keywords:** Harmonic regression, Remote sensing, Time series, Interpolated climate, Abiotic niche, Tick

## Abstract

**Background:**

Correlative modelling combines observations of species occurrence with environmental variables to capture the niche of organisms. It has been argued for the use of predictors that are ecologically relevant to the target species, instead of the automatic selection of variables. Without such biological background, the forced inclusion of numerous variables can produce models that are highly inflated and biologically irrelevant. The tendency in correlative modelling is to use environmental variables that are interpolated from climate stations, or monthly estimates of remotely sensed features.

**Methods:**

We produced a global dataset of abiotic variables based on the transformation by harmonic regression (time series Fourier transform) of monthly data derived from the MODIS series of satellites at a nominal resolution of 0.1°. The dataset includes variables, such as day and night temperature or vegetation and water availability, which potentially could affect physiological processes and therefore are surrogates in tracking the abiotic niche. We tested the capacities of the dataset to describe the abiotic niche of parasitic organisms, applying it to discriminate five species of the globally distributed tick subgenus *Boophilus* and using more than 9,500 published records.

**Results:**

With an average reliability of 82%, the Fourier-transformed dataset outperformed the raw MODIS-derived monthly data for temperature and vegetation stress (62% of reliability) and other popular interpolated climate datasets, which had variable reliability (56%–65%). The transformed abiotic variables always had a collinearity of less than 3 (as measured by the variance inflation factor), in contrast with interpolated datasets, which had values as high as 300.

**Conclusions:**

The new dataset of transformed covariates could address the tracking of abiotic niches without inflation of the models arising from internal issues with the descriptive variables, which appear when variance inflation is higher than 10. The coefficients of the harmonic regressions can also be used to reconstruct the complete original time series, being an adequate complement for ecological, epidemiological, or phylogenetic studies. We provide the dataset as a free download under the GNU general public license as well as the scripts necessary to integrate other time series of data into the calculations of the harmonic coefficients.

## Background

Various methods of species distribution modelling have been applied to arthropods of medical importance to understand the factors limiting their distributions [1-4]. These quantitative tools combine observations of species occurrence with environmental features (variously called “descriptive variables”, “environmental variables”, or “abiotic covariates”) to capture the niche of the target species and then project a prediction on a geographic range. This approach is called correlative modelling [5,6]. Such projection is generally a map illustrating the similarity of the abiotic covariates in relation to the data used to train the model. Commonly, only the abiotic component of the niche (e.g., temperature, water vapour) is used to infer the niche of the target species, although for some species, it is necessary to include an explicit description of biotic factors, like the availability of hosts, which are necessary as a blood source. These abiotic covariates are thus used to gain information about which variables may affect the fitness of the species. Because information on abiotic variables can be produced on a timely basis, correlative modelling is a useful tool for resource managers, policy makers, and scientists.

A number of modellers have argued strongly for the use of predictors that are ecologically relevant to the target species, describing the biological and ecological constraints of the species in the spatial range to be modelled [4,7-10]. However, the rule seems to be the automatic selection of variables by the modelling algorithms, relying on the statistical values of model performance [11] rather than weighting them by ecological relevance. Without such biological background, the forced inclusion of numerous variables can produce models with highly reliable matching distributions that are statistically rather than biologically relevant. The tendency in correlative modelling is to use abiotic covariates that are interpolated from climate stations [12]. These datasets describe either the monthly values of a variable (e.g., mean temperature in March) or the relationships among the variables (e.g., rainfall in the warmest quarter). The overall usefulness of these datasets for global climate studies is not in question, but they may be affected by internal issues like collinearity [13,14] that influence the reliability of the resulting spatial projection. Collinearity refers to the non-independence of predictor variables, usually in a regression-type analysis. It is a common feature of any descriptive ecological dataset and can be a problem for parameter estimation because it inflates the variance of regression parameters and hence potentially leads to the wrong identification of predictors as relevant in a statistical model [14].

Tackling the complex challenges of decision-making about human and animal health requires development of a monitoring and assessment system of the climate covering the Earth’s dimensions. Such a system must be coherent, reliable, and ready for updating as new data incorporate into the stream of observations. It ideally would supply indicators that account for climate changes and trends and how they might affect the physiological processes of the organisms to be modelled. Remotely sensed products of Earth’s processes are dynamic predictors suitable for capturing the niche preferences of some medically important arthropods [15]. Because of continuous temporal sampling, remotely sensed data provide a synoptic representation of the climate at the required spatial and temporal scales. However, the potential of such harmonised datasets to capture the abiotic niche of organisms has not yet been fully explored [16,17]. It has been mentioned that weather patterns are better surrogates for niche preferences of an organism than are the averaged and extreme values of some variables [18]. Incorporating such phenological descriptives of the abiotic niche would improve estimations of the abiotic preferences of the target organism. Studies have focused on the transformation of the time series of remotely sensed covariates via principal component analysis (PCA) or Fourier transformation [16-18]. These modifications of the time series of covariates retain the variability of the original dataset while removing the collinearity.

This paper describes a dataset of remotely sensed covariates based on the transformation by harmonic regression (time series Fourier transform) of monthly data derived from the MODIS series of satellites. Such a dataset is internally coherent, has a small number of layers to reduce the inflation of the derived models, and includes information about day and night temperature, vegetation, and water availability. This paper shows how the dataset was produced and provides the scripts necessary for further calculations. We also explicitly explored the performance of the dataset describing the abiotic niche of several species of ticks [19] and compared it with the results using other popular datasets of climate features. We provide the transformed dataset for free download under the GNU general public license serving the purpose of making specific data available to ecologists and epidemiologists.

## Methods

### A primer on harmonic regression

**Figure 1:**
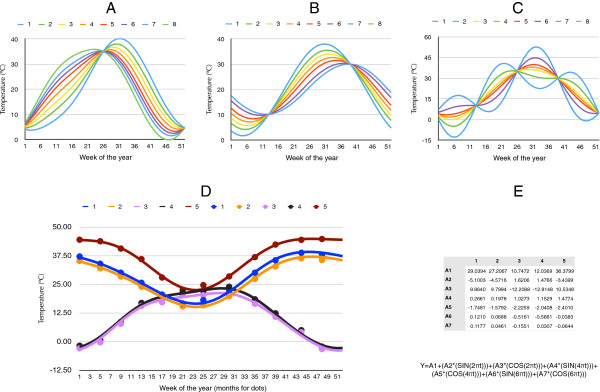
**The background of harmonic regression.** Panels **A**, **B**, and **C** show how changes in the seven coefficients of a harmonic regression (namely A1 to A7) can be used to reconstruct the mean values of a variable and the peak moment of the year can be modelled. In A, the pattern is obtained leaving A1 = 20, A3 = −15, A4 = 2.357, A5 = −0.12, A6 = −0.094, and A7 = −0.237. The value of A2 was varied between −10 and 10 at constant intervals to produce the pattern observed in the series 1–8. In B, values were left constant for A1 (20) A3 (−10) and A4 to A7 (−0.12), while the value of A3 was varied between −15 and −1, at constant intervals to produce the pattern reproduced. It is observed that changes in A2 and A3 account for the seasonality of the complete year, showing the peak of a variable in both its value and moment of the year. In C, A4 was varied between −15 and 15 at constant intervals leaving the other coefficients with fixed values, namely A1 = 20, A2 = −10, A3 = −15, A5 to A7 = −0.12. Charts in A to C show simulated temperature values. Actual data for temperature were obtained from five sites in either the northern or southern hemisphere **(D)** and then subjected to a harmonic regression (E), which was fitted with the parameters and the equation included in **E**. Capital letters in the equation refer to the rows in the table for each of the five sites simulated.

Harmonic regression is a mathematical technique used to decompose a complex signal into a series of individual sine and cosine waves, each characterised by a specific amplitude and phase angle. In the process, a series of coefficients describe the cyclical variation of the series, including its seasonal behaviour. A variable number of components can be extracted, but only a few terms are in general necessary to describe annual, semi-annual, and smaller components of the seasonal variance. In summary, the harmonic regression produces an equation with coefficients that fit the seasonal behaviour of each pixel of a series of images. When the term for time is incorporated, the coefficients reconstruct the value of the environmental variable for such time. Most important, these coefficients can be used to describe the amplitude, peak timing, seasonal peaks, seasonal threshold, and many other features of a time series [20]. Thus, harmonic regression describes the pattern of the temporal variable to be measured, from which other phenological data can be obtained. It serves as a method of potential application for capturing the abiotic niche of an organism because it describes both the pattern (seasonal components) and the ranges of climate variables between defined time intervals with the coefficients that result from the harmonic regression. The harmonic regression used in this study has the following form:

$$Y=f\left(x\right)=a_{0}+\sum_{i=1}^{n}\left(a_{i}\cos\frac{\mathrm{n\pi x}}{L}+b_{i}\sin\frac{\mathrm{n\pi x}}{L}\right)$$

where *Y* is the value of the variable at a moment of the year, α_0_ is the offset, *ai* is the coefficient of the *i*th oscillation, *L* is the fundamental frequency, and *x* is the time-dependent variable. The coefficients of the harmonic regression are referred to here as “environmental covariates” because they explicitly represent the environmental niche that an organism may occupy. The final form of the regression equation is Y = A + (B*(sin(2πt))) + (C*(cos(2πt))) + (D*(sin(4πt))) + (E*(cos(4πt))) + (F*(sin(6πt))) + (G*(cos(6πt))) where A, B, C, D, E, F, and G are the seven coefficients chosen to represent the complete time series, and *t* is the time of the year. *Y* represents the reconstructed value of a variable for the time *t*.Figure 1 displays the potential of the method to describe complex series of data. The first coefficient in the regression is the mean of the regressed variable. Each further pair of coefficients contributes to explain the complete series by determining the amplitude and the phase of periods of time that are half the length of the preceding period, e.g., twelve, six, three months, etc. Hypothetical examples in Figure 1 show how different phenological patterns are easily created, explaining the full potential of the method. Figure 1D displays real monthly values of temperature, randomly selected from two sites in the northern and southern hemispheres, compared with the weekly reconstruction of these actual series using the equation and the coefficients in Figure 1E, where “t” is the time of the year. The error of the fitted equations to the actual data is less than 1%, as measured by the residuals.

The interest of harmonic regression is that a few coefficients are able to reconstruct even daily values of the target variable (weekly in the example of Figure 1D). We claim that these coefficients retain the ecological meaning of the variable, because after reconstruction of the time series, standard features (in terms of “length of the summer”, “peak of humidity in spring” or “number of days below 0°C”) are still available using simple algebra [20]. The reduction of the time series by other methods, like Principal Components, allows the destruction of such seasonal component [21]. In correlative modelling, harmonic regression defines the abiotic niche with a few variables, therefore improving the reliability of the models because internally correlated variables, like time series, are not included [21].

### The series of data

All the data were obtained from the NEO’s (NASA Earth Observations) web server (http://neo.sci.gsfc.nasa.gov/about/). The mission of NEO is to provide an interface to browse and download satellite data from NASA’s constellation of Earth Observing System satellites. Over 50 different global datasets are represented with daily, weekly, and monthly snapshots. NEO is part of the EOS Project Science Office located at the NASA Goddard Space Flight Center.

Four series of data were targeted because of their potential to describe the abiotic niche of parasitic organisms: the Land Surface Temperature, either at day or night (LSTD, LSTN); the Normalised Difference Vegetation Index (NDVI); and the Leaf Area Index (LAI). The first expresses the temperature at the ground surface with a precision of one decimal. We worked out both LSTD and LSTN because the phenological curve of these datasets can address calculations of the total accumulated temperature over a given threshold, which is important in the detection of habitat. The NDVI is a measure of the photosynthetic activity of plants. Its value has been proven in the field of large-scale monitoring of vegetation cover, and it has been extensively used as a descriptive variable of the habitat for medically important arthropods [22,23]. NDVI thus represents an adequate source of data to cope with the water component of the arthropod life cycle, assessing temporal aspects of vegetation development and quality [23,24]. However, the relationship between NDVI and vegetation can be biased in low-vegetated areas, unless the soil background is taken into account [25]. The LAI defines an important structural property of a plant canopy, the number of equivalent layers of leaf vegetation relative to a unit of ground area [26]. This feature is important for the abiotic niche of an organism because it measures how the ground is protected against the sun and its evaporative capacities.

The four series of covariates (LSTD, LSTN, NDVI, and LAI) were obtained from the NEO website at a resolution of 0.1°, from October 2000 to December 2012 at 8-day intervals. The available sets of images have been already processed by the MODIS team, with improved cloud masking and adequate atmospheric correction and satellite orbital drift correction applied. Such processing is extremely important because the raw data are free of pixels contaminated by clouds or ice, which avoids interpretation errors. We prepared one month composites from the 8-day images, using the method of the maximum pixel value, to obtain the largest area without gaps in pixels. Data were filtered using a Savitzky–Golay smoothing filter [27]. One of the problems with applying remotely sensed imagery to the detection of abiotic niche is the existence of gaps at regions near the poles because of the long-lasting accumulation of snow, ice, or clouds. The effects are larger in the northern hemisphere because of the proximity of inhabited lands to the North Pole. The detection of these gaps and filling them with estimated values may be unreliable if the number of consecutive gaps is too long [28]. Some regions in the far North were not included in the final set of images because they were covered by snow, clouds, or ice for periods longer than 4 months.

Monthly values of each variable were subjected to harmonic regression. We performed the harmonic regressions in the R development framework [29] together with the packages “raster” [30] and “TSA” [31]. Seven coefficients for each variable were extracted from the annual time series. A script is provided as Additional file 1, illustrating the production of the coefficients of the harmonic regression. The coefficients representing the yearly, 6-month, and 3-month signals were selected from the harmonic regressions. Thus, seven layers of coefficients of each variable could reconstruct the complete original time series and constitute the environmental covariates proposed in this paper to describe the abiotic niche of organisms.

A RGB composition of the four sets of harmonic coefficients is included in Additional file 2: Figure S1.

### Comparison of performance of the environmental variables

We aimed to demonstrate that (i) the coefficients of the harmonic regression have a significantly smaller collinearity than the original MODIS-derived time series and other popular climate datasets commonly used in correlative modelling, and (ii) that the performance of the harmonic coefficients in describing the abiotic niche of parasitic organisms is better than other products commonly used for this purpose. Collinearity is a statistical phenomenon of a dataset of spatial covariates [14]. Two or more variables in a multiple regression model may be highly correlated and then inflate the reliability of the model. In our application, the typical situation involves the use of time series of covariates that are strongly correlated (e.g., the temperature in one month is expected to be very similar to the values of the following month). A special situation exists when covariates are grid interpolations of climate point records. In this case, the problems are magnified because the interpolation algorithms use a set of discrete, irregularly spaced sites (the meteorological stations) and the temporal series of covariates will exhibit a high collinearity. We assessed collinearity of the covariates with the variance inflation factor (VIF), which is a measure of correlation between pairs of variables [32]. Values of VIF > 10 denote a potentially problematic collinearity within the set of covariates, indicating that these covariates should be removed from model development [33]. A VIF = 1 indicates that the variables are orthogonal. VIF was calculated with the package “fmsb” [34] for R on the monthly values of LSTD, LSTN, NDVI, and LAI, as well as the derived harmonic coefficients. To compare with other popular products used in the inference of the abiotic niche, we computed the VIF of the monthly values of temperature and rainfall of Worldclim (http://www.worldclim.org) and the so-called “bioclimate variables” from the same source, which are calculated ratios among some significant variables [35] at the same spatial resolution as the remotely sensed data.

**Figure 2:**
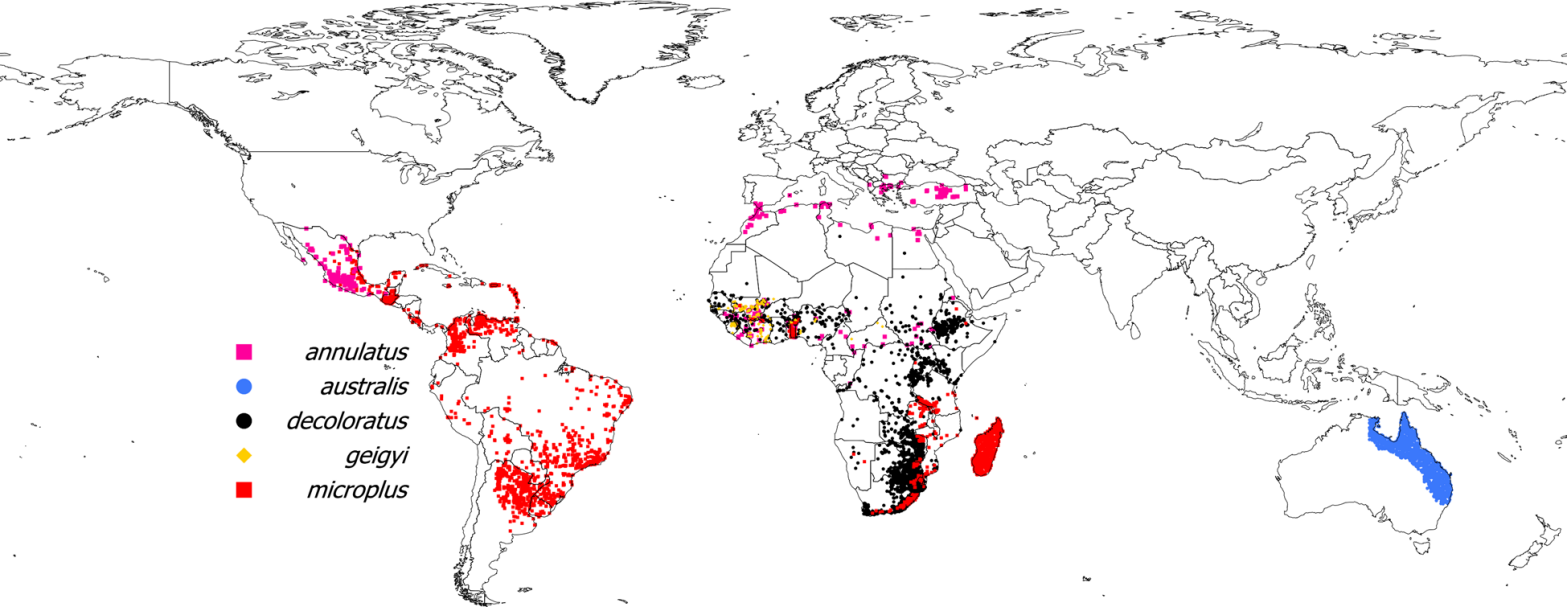
**The reported distribution of 9,534 records of ticks of the subgenus *Boophilus*.** Only records with a pair of coordinates were included in the map and considered for further computations. Records from Asia lack such reliable georeferencing and were not included.

The performance of the models built with these abiotic covariates was tested on a dataset of the reported world distribution of ticks of the subgenus *Boophilus*. This database of tick distribution has a global extent and is therefore appropriate for an explicit test of the environmental covariates. These ticks have a recent history of introduction by the trade movements of livestock [19], and some species are sympatric and thus may have similar preferences for defined portions of the abiotic niche [36]. Thus, the reported world distribution of boofilid ticks is a demanding statistical problem of discrimination among species because some of them may share a portion of the available ecological niche. We used the known distribution data for *Rhipicephalus (B.) annulatus*, *R. australis*, *R. decoloratus*, *R. geigyi*, and *R. microplus*, which consists of 9,534 records for the five species. Few details are known about the distribution of *R. kohlsi*, and it was removed from further calculations. Details of the compilation of the original dataset have been provided [36], but the dataset has been updated with new records from Africa and South America published after the date of the original compilation. Figure 2 shows the spatial distribution of the world records of the five species.

We wanted to discriminate among the five species of ticks as a proof of concept, using different datasets. This application is intended to allow inferences regarding the abiotic conditions behind an observed distribution of an organism, not to project such inferences onto the spatial domain but to correctly classify the set of records. The best set of abiotic covariates will produce the best description of the abiotic niche of these species of ticks, thus allowing the best discrimination among species. We built a discriminant analysis with the records of the five species of ticks and the different datasets of environmental covariates. Details of the discriminant analysis approach to distribution models or epidemiological issues have been addressed elsewhere [37,38]. We used a standard (linear) approach to the discriminant analysis, which uses a common (within-) covariance matrix for all groups. We used stepwise variable selection to control which variables are included in the analysis. We used the discriminant scores, the distance to the mean of that classification, and the associated probability to assign the classification of each record of ticks included in this study. The performance of such models is traditionally assessed by calculating the area under the curve (AUC) of the receiver operator characteristic [39], a plot of the sensitivity (the proportion of correctly predicted known presences, also known as absence of omission error) vs. 1 – specificity (the proportion of incorrectly predicted known absences or the commission error) over the whole range of threshold values between 0 and 1. The model AUC thus calculated is compared to the null model that is an entirely random predictive model with AUC = 0.5, and models with an AUC above 0.75 are normally considered useful [40]. Using this method, the commission and omission errors are therefore weighted with equal importance for determining the performance of the model. Other than the calculation of AUC, we explicitly evaluated the percentage of correctly determined records of ticks, using the different sets of abiotic covariates.

To capture the abiotic niche and thus discriminate the five species of ticks, we used (i) the coefficients of the harmonic regression of LSTD and NDVI; (ii) the same set of (i) plus the coefficients of the harmonic regression of LAI; (iii) remotely sensed monthly averages of LSTD and NDVI; (iv) the same set in (iii) after removal of the pairs of covariates with VIF > 10; (v) monthly averages of temperature and rainfall obtained from Worldclim; (vi) bioclimate variables from the Worldclim dataset; and (vii and viii) monthly Worldclim values and bioclimate variables after removal of the covariates with VIF > 10, respectively. No attempts were made to include LSTN in these efforts because it parallels the phenology of LSTD. We are aware that NDVI is not highly correlated with rainfall, but it is commonly used as a surrogate of drought conditions [41], and its performance can therefore be compared with rainfall estimates.

## Results

Table 1 includes the collinearity values among the seven coefficients of the harmonic regressions of each series of remotely sensed covariates over the complete Earth’s surface. The calculation of collinearity between LSTD and LSTN was omitted because they express the same variable either at day or night and are obviously highly correlated. The collinearity among the harmonic environmental variables was lower than 3 for every possible combination, an indication that all of these covariates could be used together to train models without inflation of the resulting inference. However, the monthly series of remotely sensed covariates had values of VIF higher than 200 (Tables 2, 3 and 4), and the maximum statistically allowable is around 10. The transformation of the monthly series of remotely sensed covariates removes the collinearity while retaining its complete ecological meaning. Tables 5 and 6 show the VIF values for the monthly series of interpolated temperature and rainfall, respectively. A total of 45% of monthly combinations of temperature and 6% of monthly combinations of rainfall produced VIF values higher than 10. The “bioclim” variables were also affected by the collinearity (Table 7). Some combinations of these covariates produced high VIF values, including combinations of variables related to temperature (e.g., annual mean, mean of coldest quarter, seasonality, annual range, maximum and mean of warmest quarter, minimum and mean of driest quarter) and a few combinations of rainfall (wettest period and quarter and driest period and quarter) that are intuitively correlated.

**Table 1:** Collinearity among the coefficients of the harmonic regression of T, NDVI, and LAI

	**T1**	**T2**	**T3**	**T4**	**T5**	**T6**	**T7**	**NDVI1**	**NDVI2**	**NDVI3**	**NDVI4**	**NDVI5**	**NDVI6**	**NDVI7**	**LAI1**	**LAI2**	**LAI3**	**LAI4**	**LAI5**	**LAI6**
T2	1.03																			
T3	1.85	1.20																		
T4	1.00	1.03	1.00																	
T5	1.37	1.02	1.01	1.07																
T6	1.02	1.00	1.00	1.00	1.02															
T7	1.37	1.02	1.26	1.02	1.07	1.00														
NDVI1	1.01	1.00	1.17	1.00	1.00	1.00	1.02													
NDVI2	1.05	1.29	1.02	1.04	1.00	1.00	1.01	1.00												
NDVI3	3.00	1.15	1.85	1.04	1.08	1.02	1.33	1.01	1.16											
NDVI4	1.00	1.00	1.01	1.03	1.04	1.07	1.01	1.06	1.04	1.01										
NDVI5	1.59	1.01	1.20	1.01	1.32	1.01	1.28	1.11	1.02	1.48	1.19									
NDVI6	1.28	1.01	1.11	1.01	1.04	1.01	1.22	1.00	1.03	1.40	1.02	1.09								
NDVI7	1.05	1.00	1.02	1.00	1.13	1.00	1.00	1.07	1.00	1.01	1.22	1.27	1.04							
LAI1	1.00	1.00	1.07	1.01	1.23	1.00	1.01	3.18	1.00	1.02	1.00	1.01	1.00	1.01						
LAI2	1.19	1.07	1.16	1.00	1.00	1.00	1.17	1.01	1.45	1.08	1.06	1.01	1.10	1.01	1.03					
LAI3	1.43	1.22	1.22	1.01	1.02	1.01	1.00	1.01	1.19	2.28	1.07	1.03	1.07	1.00	1.00	1.00				
LAI4	1.40	1.01	1.16	1.05	1.02	1.00	1.17	1.00	1.01	1.26	1.18	1.01	1.30	1.03	1.00	1.61	1.11			
LAI5	1.21	1.02	1.10	1.00	1.06	1.00	1.02	1.00	1.03	1.29	1.00	1.36	1.03	1.02	1.01	1.00	1.45	1.01		
LAI6	1.11	1.01	1.02	1.01	1.01	1.01	1.05	1.00	1.00	1.07	1.00	1.03	1.40	1.00	1.00	1.05	1.07	1.36	1.06	
LAI7	1.00	1.00	1.00	1.00	1.00	1.01	1.02	1.00	1.00	1.00	1.01	1.04	1.00	1.13	1.00	1.02	1.00	1.03	1.08	1.00

Collinearity was calculated as the variance inflation factor. Values lower than 10 are indicative of low collinearity and could be used together in models of the environmental niche. The number after the letters of the variables indicates the ordinal coefficient in the harmonic regression of the variable.

**Table 2:** Collinearity among the monthly values of temperature

	**Jan**	**Feb**	**Mar**	**Apr**	**May**	**Jun**	**Jul**	**Aug**	**Sep**	**Oct**	**Nov**
Feb	58.43										
Mar	9.59	20.80									
Apr	3.77	5.51	18.89								
May	2.18	2.75	5.32	19.04							
Jun	1.59	1.85	2.80	5.58	20.08						
Jul	1.63	1.89	2.82	5.42	16.42	142.31					
Aug	1.66	1.93	2.90	5.60	16.29	69.95	229.85				
Sep	2.55	3.21	6.14	17.01	30.26	12.84	14.47	18.06			
Oct	4.58	6.62	18.71	32.47	10.29	4.50	4.67	5.10	19.31		
Nov	16.60	31.02	33.66	8.83	3.73	2.28	2.34	2.43	4.82	14.85	
Dec	213.04	56.23	10.83	4.13	2.32	1.66	1.70	1.74	2.75	5.25	23.96

Eight-day-interval MODIS-derived series of values were converted to monthly composites and collinearity calculated as the variance inflation factor. Values higher than 10 are indicative of high collinearity.

**Table 3:** Collinearity among the monthly values of the normalised difference vegetation index

	**Jan**	**Feb**	**Mar**	**Apr**	**May**	**Jun**	**Jul**	**Aug**	**Sep**	**Oct**	**Nov**
Feb	35.82										
Mar	15.40	31.65									
Apr	5.09	6.05	10.50								
May	1.74	2.04	2.31	4.27							
Jun	1.13	1.23	1.27	1.53	3.75						
Jul	1.06	1.10	1.13	1.25	2.09	11.21					
Aug	1.11	1.16	1.18	1.32	2.21	7.43	24.85				
Sep	1.59	1.75	1.81	2.33	4.36	3.75	3.06	4.55			
Oct	2.54	2.66	2.80	4.26	4.67	1.97	1.57	1.88	7.74		
Nov	6.11	4.97	5.50	7.58	2.87	1.37	1.20	1.33	2.87	8.89	
Dec	31.42	12.00	9.80	4.95	1.79	1.22	1.14	1.20	1.75	2.79	9.14

Eight-day-interval MODIS-derived series of values were converted to monthly composites and collinearity calculated as the variance inflation factor. Values higher than 10 are indicative of high collinearity.

**Table 4:** Collinearity among the monthly values of the leaf area index

	**Jan**	**Feb**	**Mar**	**Apr**	**May**	**Jun**	**Jul**	**Aug**	**Sep**	**Oct**	**Nov**
Feb	3.21										
Mar	2.14	7.88									
Apr	2.23	5.26	9.48								
May	1.89	3.23	4.38	6.35							
Jun	1.50	2.86	3.68	4.91	18.29						
Jul	1.21	2.84	3.64	4.82	16.73	54.59					
Aug	1.56	2.89	3.72	4.94	17.06	43.16	62.82				
Sep	1.98	3.00	3.91	5.29	21.09	37.14	38.85	50.07			
Oct	2.01	5.54	7.93	9.71	6.13	4.85	4.77	4.91	5.27		
Nov	2.92	6.72	6.36	4.78	3.08	2.75	2.73	2.78	2.88	5.08	
Dec	3.05	4.17	3.21	2.71	2.14	2.00	1.99	2.01	2.06	2.74	4.76

Eight-day-interval MODIS-derived series of values were converted to monthly composites and collinearity calculated as the variance inflation factor. Values higher than 10 are indicative of high collinearity.

**Table 5:** Collinearity among the monthly values of temperature obtained by interpolated data (Worldclim)

	**Jan**	**Feb**	**Mar**	**Apr**	**May**	**Jun**	**Jul**	**Aug**	**Sep**	**Oct**	**Nov**
Feb	184.56										
Mar	24.31	50.92									
Apr	7.77	10.89	32.00								
May	3.61	4.34	7.17	21.47							
Jun	1.91	2.11	2.75	4.52	12.87						
Jul	1.50	1.61	1.96	2.77	5.24	27.31					
Aug	1.88	2.06	2.64	4.07	8.91	37.03	38.90				
Sep	3.63	4.27	6.59	13.49	27.88	10.75	5.80	12.66			
Oct	10.07	13.78	29.90	40.82	12.22	3.91	2.61	3.97	15.91		
Nov	41.60	58.81	45.65	14.48	5.40	2.42	1.81	2.43	5.69	27.95	
Dec	329.62	152.40	27.89	8.74	3.89	2.00	1.56	1.98	3.99	12.30	73.92

Collinearity was calculated as the variance inflation factor. Values higher than 10 are indicative of high collinearity.

**Table 6:** Collinearity among the monthly values of rainfall obtained by interpolated data (Worldclim)

	**Jan**	**Feb**	**Mar**	**Apr**	**May**	**Jun**	**Jul**	**Aug**	**Sep**	**Oct**	**Nov**
Feb	24.41										
Mar	6.82	12.22									
Apr	2.12	2.54	4.86								
May	1.23	1.29	1.57	3.33							
Jun	1.03	1.04	1.10	1.40	3.58						
Jul	1.00	1.00	1.02	1.14	1.76	5.42					
Aug	1.00	1.00	1.02	1.13	1.72	4.14	13.64				
Sep	1.05	1.05	1.11	1.35	2.23	3.55	3.34	5.06			
Oct	1.38	1.37	1.55	2.04	2.33	1.73	1.40	1.53	3.09		
Nov	2.70	2.40	2.65	2.47	1.59	1.16	1.06	1.08	1.34	3.55	
Dec	10.33	6.13	4.56	2.24	1.30	1.05	1.01	1.01	1.10	1.73	5.77

Collinearity was calculated as the variance inflation factor. Values higher than 10 are indicative of high collinearity.

**Table 7:** Collinearity among the “bioclim” variables derived from interpolated data

	**Bio1**	**Bio2**	**Bio3**	**Bio4**	**Bio5**	**Bio6**	**Bio7**	**Bio8**	**Bio9**	**Bio10**	**Bio11**	**Bio12**	**Bio13**	**Bio14**	**Bio15**	**Bio16**	**Bio17**	**Bio18**
Bio2	1.34																	
Bio3	3.40	1.16																
Bio4	3.29	1.04	4.79															
Bio5	5.05	1.96	1.62	1.36														
Bio6	15.65	1.13	4.72	8.17	2.40													
Bio7	2.15	1.00	3.10	17.21	1.15	4.31												
Bio8	2.98	1.38	1.71	1.35	3.54	2.01	1.18											
Bio9	8.46	1.22	2.90	3.91	2.68	10.14	2.58	1.61										
Bio10	8.08	1.59	1.80	1.53	41.52	3.15	1.27	4.04	3.27									
Bio11	25.25	1.20	4.84	7.05	2.72	123.00	3.53	2.18	10.45	3.60								
Bio12	1.18	1.07	1.48	1.46	1.02	1.33	1.62	1.08	1.18	1.05	1.28							
Bio13	1.28	1.01	1.52	1.48	1.06	1.40	1.54	1.17	1.22	1.11	1.37	5.14						
Bio14	1.01	1.16	1.06	1.07	1.02	1.04	1.15	1.00	1.02	1.00	1.02	2.06	1.20					
Bio15	1.15	1.35	1.08	1.03	1.22	1.07	1.00	1.25	1.07	1.20	1.11	1.03	1.02	1.36				
Bio16	1.26	1.02	1.52	1.49	1.05	1.39	1.56	1.15	1.21	1.10	1.36	6.72	69.34	1.24	1.01			
Bio17	1.01	1.16	1.08	1.09	1.01	1.05	1.18	1.00	1.02	1.00	1.03	2.29	1.24	84.01	1.36	1.29		
Bio18	1.06	1.04	1.16	1.14	1.00	1.10	1.19	1.08	1.03	1.01	1.09	2.80	2.24	1.48	1.01	2.39	1.54	
Bo19	1.07	1.06	1.24	1.21	1.00	1.16	1.30	1.01	1.11	1.02	1.12	2.36	1.54	1.85	1.08	1.61	1.98	1.18

Collinearity was calculated as the variance inflation factor. Values higher than 10 are indicative of high collinearity. The names Bio1 to Bio19 are the names defined by the Worldclim dataset, namely annual mean temp., mean diurnal range, isothermality, temp. seasonality, max temp. of warmest month, min. temp. of coldest month, temp. annual range, mean temp. of wettest quarter, mean temp. of driest quarter, mean temp. of warmest quarter, mean temp. of coldest quarter, annual precipitation, precipitation of wettest month, precipitation of driest month, precipitation seasonality, precipitation of wettest quarter, precipitation of driest quarter, precipitation of warmest quarter, precipitation of coldest quarter.

Table 8 reports the results of the discriminant analysis trained with different combinations of environmental covariates applied to the dataset of the world distribution of the ticks of the subgenus *Boophilus*. The table includes data on both the percentage of records correctly identified by each model and the AUC values, a measure of general reliability. All the models performed variably, but the best overall performance was obtained for the Fourier-derived covariates including seven coefficients of LSTD and NDVI and the first five coefficients of LAI, with 82.4% correct determinations. This model produced the best discrimination between *R. annulatus* and *R. geigyi*, with almost 70% of records of the former correctly determined. The performance of discriminant analysis decreased if only the seven coefficients of LSTD and NDVI were included (14 covariates, 72.9% of correct determinations). Models trained with the monthly series of LSTD and NDVI (24 partially correlated variables) had poorer performance (62.3% of correct determinations), which further decreased after removal of covariates with high VIF (12 variables, 56.7% of correct determinations). Discriminant models built with 24 covariates of gridded interpolated data of temperature and rainfall performed slightly better than remotely sensed covariates (69.7%). Such performance decreased when pairs of covariates with high VIF were removed (16 covariates, 65.1%). It is interesting to note the low overall performance of the discriminant analysis trained with 19 covariates derived from the interpolated climate, the so-called “bioclim” variables (57.9%), which further decreased after removal of the pairs of covariates showing high VIF (7 variables, 57.4%). The low discriminant capacity of such a set of derived interpolated covariates can be observed comparing the slight differences in performance if covariates with high VIF are removed from the model training: There was only a drop of 0.5% of correctly determined records after the removal of as many as 12 variables. With this application, the “bioclim” dataset had the poorest performance in capturing the abiotic niche of the set of records of the world distribution of boofilid ticks.

**Table 8:** **Percent of correctly discriminated species of the subgenus *Boophilus*, using the sets of descriptive covariates**

**Records reported as**	**AUC**	***annulatus***	***australis***	***decoloratus***	***geigyi***	***microplus***
1. Discriminant analysis with 7 coefficients of LST, 7 coefficients of NDVI, and 5 coefficients of LAI. Correct determinations: 82.4%						
*annulatus*	0.955	69.94	0.00	2.76	25.15	2.15
*australis*	0.977	0.00	93.39	1.17	0.00	5.43
*decoloratus*	0.905	2.21	0.18	79.85	9.26	8.50
*geigyi*	0.986	1.41	0.00	1.41	97.18	0.00
*microplus*	0.924	2.53	1.86	16.39	1.31	77.91
2. Discriminant analysis with 7 coefficients of LST, and 7 coefficients of NDVI. Correct determinations: 72.9%						
*annulatus*	0.959	46.79	0.00	3.21	48.08	1.92
*australis*	0.995	0.02	94.45	1.44	0.00	4.08
*decoloratus*	0.922	4.94	0.09	73.84	8.94	12.19
*geigyi*	0.989	3.47	0.00	1.39	93.75	1.39
*microplus*	0.946	5.36	1.33	12.06	0.73	80.52
3. Discriminant analysis with 12 months of remotely sensed LST and NDVI. Correct determinations: 62.3%						
*annulatus*	0.931	32.69	1.28	4.49	57.05	4.49
*australis*	0.991	0.20	96.83	1.22	0.00	1.75
*decoloratus*	0.889	4.27	1.91	69.93	10.77	13.12
*geigyi*	0.979	6.25	0.69	0.00	92.36	0.69
*microplus*	0.919	4.68	5.93	16.69	2.62	70.08
4. Discriminant analysis with monthly remotely sensed LST and NDVI, after removal of months with high collinearity. Only values for January, March, May and October were included for LST. Data for February, March and July were removed from NDVI. Correct determinations: 56.7%						
*annulatus*	0.912	34.62	1.28	0.64	57.05	6.41
*australis*	0.947	0.49	90.83	3.20	0.00	5.48
*decoloratus*	0.761	5.12	12.06	52.89	14.10	15.84
*geigyi*	0.971	9.72	1.39	0.69	88.19	0.00
*microplus*	0.826	8.23	15.12	18.87	2.46	55.32
5. Discriminant analysis with 12 months of gridded interpolated temperature and rainfall (Worldclim dataset). Correct determinations: 69.7%						
*annulatus*	0.872	42.31	1.92	3.85	44.23	7.69
*australis*	0.996	0.00	99.67	0.13	0.00	0.20
*decoloratus*	0.923	4.23	1.33	78.29	7.96	8.19
*geigyi*	0.985	5.56	0.69	2.78	85.42	5.56
*microplus*	0.949	2.66	7.78	14.88	0.81	73.87
6. Discriminant analysis with monthly gridded interpolated temperature and rainfall (Worldclim dataset) after removal of the months with high collinearity. Only data of temperatures of January, April, June and September were included. Data of rainfall of February, August and December were removed. Correct determinations: 65.1%						
*annulatus*	0.889	44.23	4.49	0.64	45.51	5.13
*australis*	0.982	0.00	97.63	2.35	0.00	0.02
*decoloratus*	0.851	8.41	3.65	72.15	10.63	5.16
*geigyi*	0.965	15.97	0.00	0.00	76.39	7.64
*microplus*	0.879	6.45	16.57	14.88	3.06	59.03
7. Discriminant analysis with “bioclim variables” derived from monthly gridded interpolated temperature and rainfall (Worldclim dataset). Correct determinations: 57.9%						
*annulatus*	0.941	28.21	1.92	8.97	46.15	14.74
*australis*	0.990	0.00	97.45	0.00	0.00	2.55
*decoloratus*	0.876	3.91	2.58	74.64	9.34	9.52
*geigyi*	0.968	1.39	2.08	3.47	83.33	9.72
*microplus*	0.901	1.49	11.09	19.60	1.98	65.85
8. Discriminant analysis with “bioclim variables” derived from monthly gridded interpolated temperature and rainfall (Worldclim dataset) after removal of variables with high collinearity. Correct determinations: 57.4%						
*annulatus*	0.930	29.49	1.92	7.69	53.21	7.69
*australis*	0.991	0.00	95.12	0.00	0.09	4.79
*decoloratus*	0.890	3.51	2.49	73.84	10.85	9.30
*geigyi*	0.979	1.39	0.69	1.39	86.81	9.72
*microplus*	0.920	2.30	15.52	19.68	5.85	56.65

For some of these datasets of descriptive covariates, the analysis was repeated with every variable included (e.g., the 12 months of average temperature) and after the highly correlated variables were removed. A discriminant analysis was conducted, and its reliability evaluated by the percent of records correctly predicted and the area under the curve (AUC). The AUC is a general measure of model performance and does not consider individual results of true positives for each species. Therefore, some models may perform better for a particular species while having a general low AUC. The percent of correctly determined records of each species is also included.

## Discussion

Increased availability of species distribution and environmental datasets, combined with the development of sophisticated modelling approaches, has resulted in many recent reports evaluating the distributions of health-threatening arthropods [42-46]. This capture of the environmental niche represents an inference of the recorded distribution of the organism, which can then be projected into a different spatial or temporal framework. The capture of the abiotic niche comes with some methodological caveats, however: (i) It is necessary to select a set of descriptive covariates with an ecological meaning for the organism to be modelled [7]; (ii) these covariates must be free of statistical issues that could affect the process of inference [47]; (iii) they must cover the widest geographical range [48]; and (iv) they should be ideally prepared with the same resolution. It is commonly the case that points (i) and (ii) may be mutually exclusive, i.e., the ecologically relevant covariates are indeed highly correlated, therefore leaving only ecologically inappropriate covariates for environmental inference. The automatic selection of the covariates that render the best model, which has become popular in recently available modelling algorithms [49], introduces further unreliability in the modelling process. A large evaluation of how to deal with collinearity in environmental covariates [14] concluded that none of the purpose-built methods yielded much higher accuracies than those that ignore collinearity. As a rule, collinearity must be removed before the building of the models because it cannot be handled by further methods.

We produced a dataset of environmental variables based on the harmonic regression of remotely sensed time series of day and night temperature, vegetation stress, and leaf area index. This dataset is aimed to fit the statistical rules of internal coherence when applied to the detection of the environmental niche of organisms. Our goal was to produce a homogeneous set of uncorrelated variables, retaining the complete ecological meaning and covering the complete Earth’s surface. We obtained the raw data from a reliable source that ensures the best pre-processing, which makes for a consistent and homogeneous set of raw variables. The meaning and the potential of the harmonic regression to capture the phenology of the climate have been already pointed out [20]. We evaluated the performance of the harmonic regression coefficients with a dataset of world records of boofilid ticks, which is a challenging problem for such techniques because these species have a pan-Tropical and Mediterranean distribution [50]. In some cases, the trade movements of livestock introduced and spread species far away from the original ranges [51]. We demonstrated that the covariates derived from the harmonic regression better captured the abiotic niche of several species of ticks than did the monthly raw set of descriptors or interpolated gridded climate, which have been traditionally used for this purpose [52-54]. We are aware that the nominal spatial resolution of 0.1° may be too coarse for some applications focusing on local or regional issues, which could require a higher resolution. The choice of such resolution is a balance between complete coverage of the Earth’s surface and processing requirements in terms of time and computer resources. Such resolution is similar to a previous set focusing on remotely sensed data from the AVHRR series of sensors [55]. However, MODIS is particularly more attractive for epidemiological applications than AVHRR because of the better spectral and temporal resolutions [55].

One source of unreliability is the inference from inadequate sets of descriptive covariates, which in some cases may include a high collinearity [14]. We are considering collinearity in the context of a statistical model that is used to estimate the relationship between one response variable (the species in our application) and a set of descriptive covariates. Examples include regression models of all types, classification and regression trees, and neural networks. Coefficients of a regression can be estimated, but with inflated standard errors [56] that result in inaccurate tests of significance for the predictors, meaning that important predictors may not be significant, even if they are truly influential [14]. Extrapolation beyond the geographic or environmental range of sampled data is prone to serious errors because patterns of collinearity are likely to change. Obvious examples include use of statistical models to predict distributions of species in new geographic regions or changed climatic conditions, giving the impression of a well-fitted model to which tests of model reliability are “blind” [21,57,58].

Generalised sets of covariates produce an unmanageable level of uncertainty in species distribution models that cannot be ignored. The use of sound ecological theory and statistical methods to check predictor variables can reduce this uncertainty, but our knowledge of species may be too limited to make more than arbitrary choices. Data reduction methods are usually employed to remove these correlations and provide one or more transformed images without such correlation, which can then be used in further analyses or applications. One ordination approach commonly applied to multi-temporal imagery is PCA [59], but explicit measures of seasonality are lost in the ordination process. PCA thus achieves data reduction at the expense of biological descriptiveness. Alternative methods that retain information about seasonality include polynomial functions [10] and temporal Fourier analysis [17,18]. The Fourier transformation of remotely sensed variables has been proposed as a reliable approach to define the niche of organisms [18,19,60] because it retains the complete variability of the original time series as well as the ecological meaning. Temporal harmonic regression transforms a series of observations taken at intervals over a period of time into a set of (uncorrelated) sine curves, or harmonics, of different frequencies, amplitudes, and phases that collectively sum to the original time series. A high-resolution version of AVHRR data converted into Fourier derivate, focused on the western Palearctic, was made available commercially [54], and a general algorithm to handle MODIS images and decompose them into harmonics was already available [18]. Our application is thus the first to provide a set of statistically suitable, internally coherent set of variables with ecological meaning, aimed at describing the abiotic niche of organisms and covering the complete Earth’s surface. While this new set of environmental descriptors has been developed to delineate the associations of parasites with abiotic traits and how these traits can shape potential distributions, it would potentially benefit ecologists and epidemiologists in the capture of the abiotic niche of other organisms.

## Conclusions

The set of environmental covariates described in this study covers the complete Earth and lacks internal issues that may inflate the models derived. It targets capturing the abiotic niche of organisms, with potential applications in a variety of fields in ecology, epidemiology, and phylogeography. The tests, applied to a worldwide collection of records of five species of ticks with overlapping spatial distributions, demonstrated that the environmental variables derived from a harmonic regression better discriminated the species, and therefore their abiotic niche, outperforming the reliability of other sets of environmental covariates and not inflating the models as a result of the collinearity of the descriptors, which were measured by the VIF. The usefulness of interpolated gridded covariates is not in question in many fields, but it must be stressed that they offer limited value for describing the abiotic niche of ticks because the application of statistical rules may force removal of ecologically relevant covariates describing such a niche. We have made the set of coefficients of the harmonic regressions available for free download and provided the scripts necessary to either reproduce the workflow or to apply the methodology to new sets of time variables.

## Abbreviations

LAI: Leaf area index; LSTD: Land surface temperature (day); LSTN: Land surface temperature (night); NDVI: Normalised difference vegetation index; PCA: Principal components analysis; VIF: Variance inflation factor.

## Competing interests

The authors declare that they have no competing interests.

## Authors’ contributions

AEP conceived the study, AES processed the images, and all authors analysed the results. AEP and JF wrote the paper. All authors read and approved the final manuscript.

## Supplementary Material

Additional file 1 **Script Fourier.** This is an R script to ingest the time series of remotely sensed images and obtain the coefficients of the harmonic regression. Brief instructions are provided as comments in the script. Click here for file

Additional file 2: Figure S1 Composite images of the coefficients of harmonic regression for the four series of remotely sensed covariates. Composites represent LSTD (A), LSTN (B), NDVI (C), and LAI (D). Compositions related to LSTD and LSTN were prepared with A1 (red), A2 (blue), and A3 (green) coefficients (i.e., the three first coefficients of the harmonic regression for each variable). Compositions regarding NDVI and LAI were prepared with the A1 (green), A2 (blue), and A3 (red) coefficients. Click here for file

## References

[B1] Kalluri S Gilruth P Rogers D Szczur M Surveillance of arthropod vector-borne infectious diseases using remote sensing techniques: a review PLoS Pathog 2007 3 e116 10.1371/journal.ppat.0030116 PMC2042005 17967056

[B2] Diuk-Wasser MA Brown HE Andreadis TG Fish D Modeling the spatial distribution of mosquito vectors for West Nile virus in Connecticut, USA Vector-Borne & Zoonotic Dis 2006 6 283 295 10.1089/vbz.2006.6.283 16989568

[B3] Estrada-Peña A Venzal JM Climate niches of tick species in the Mediterranean region: modeling of occurrence data, distributional constraints, and impact of climate change J Med Entomol 2007 44 1130 1138 18047215 10.1603/0022-2585(2007)44[1130:cnotsi]2.0.co;2

[B4] Cumming GS Van Vuuren DP Will climate change affect ectoparasite species ranges? Global Ecol Biogeog 2006 15 486 497

[B5] Elith J Kearney M Phillips S The art of modelling range-shifting species Meth Ecol Evol 2010 1 4 330 342

[B6] Kearney MR Wintle BA Porter WP Correlative and mechanistic models of species distribution provide congruent forecasts under climate change Conserv Lett 2010 3 203 213

[B7] Randolph SE Tick ecology: processes and patterns behind the epidemiological risk posed by ixodid ticks as vectors Parasitol 2004 129 S37 S65 10.1017/s0031182004004925 15938504

[B8] Glass GE Schwartz BS Morgan JM Johnson DT Noy PM Israel E Environmental risk factors for Lyme disease identified with geographic information systems Am J Public Hlth 1995 85 944 948 10.2105/ajph.85.7.944 PMC1615529 7604918

[B9] Guerra M Walker E Jones C Paskewitz S Cortinas MR Stancil A Beck L Bobo M Kitron U Predicting the risk of Lyme disease: habitat suitability for *Ixodes scapularis* in the north central United States Emerg Infect Dis 2002 8 289 297 11927027 10.3201/eid0803.010166 PMC2732460

[B10] Ogden NH Barker IK Beauchamp G Brazeau S Charron DF Maarouf A Morshed MG O’Callaghan CJ Thompson RA Waltner-Toews D Waltner-Toews M Lindsay LR Investigation of ground level and remote-sensed data for habitat classification and prediction of survival of *Ixodes scapularis* in habitats of southeastern Canada J Med Entomol 2009 43 403 414 16619627 10.1603/0022-2585(2006)043[0403:ioglar]2.0.co;2

[B11] Segurado P Araujo MB An evaluation of methods for modelling species distributions J Biogeog 2004 31 1555 1568

[B12] Kriticos DJ Webber BL Leriche A Ota N Macadam I Bathols J Scott JK CliMond: global high‒resolution historical and future scenario climate surfaces for bioclimatic modelling Methods Ecol Evol 2012 3 53 64

[B13] Douglass DH Clader BD Christy JR Michaels PJ Belsley DA Test for harmful collinearity among predictor variables used in modeling global temperature Climate Res 2003 24 15 18

[B14] Dormann CF Elith J Bacher S Buchmann C Carl G Carré G Lautenbach S Collinearity: a review of methods to deal with it and a simulation study evaluating their performance Ecography 2013 36 27 46

[B15] Hay SI Snow RW Rogers DJ From predicting mosquito habitat to malaria seasons using remotely sensed data: practice, problems and perspectives Parasitoly Today 1998 14 306 313 10.1016/s0169-4758(98)01285-x 17040796

[B16] Green RM Hay SI The potential of Pathfinder AVHRR data for providing surrogate climatic variables across Africa and Europe for epidemiological applications Remote Sens Environ 2002 79 166 175 22581983 10.1016/S0034-4257(01)00270-X PMC3350066

[B17] Scharlemann JP Benz D Hay SI Purse BV Tatem AJ Wint GW Rogers DJ Global data for ecology and epidemiology: a novel algorithm for temporal Fourier processing MODIS data PLoS One 2008 3 e1408 18183289 10.1371/journal.pone.0001408 PMC2171368

[B18] Rogers DJ Hay SI Packer MJ Predicting the distribution of tsetse flies in West Africa using temporal Fourier processed meteorological satellite data Annals Trop Med Parasitol 1996 90 225 242 10.1080/00034983.1996.11813049 8758138

[B19] de León AA P Strickman DA Knowles DP Fish D Thacker E de la Fuente J Krause P Wikel SK Miller RS Wagner GG Almazán C Hillman R Messenger MT Ugstad PO Duhaime RA Teel PD Ortega-Santos A Hewitt DG Bowers EJ Bent S Cochran MH McElwain TF Scoles GA Suarez CE Davey R Howell Freeman JM Lohmeyer K Li A Guerrero F Kammlah D One health approach to identify research needs in bovine and human babesioses: Workshop report Parasites and Vectors 2010 3 36 20377902 10.1186/1756-3305-3-36 PMC2859369

[B20] Lofgren E Fefferman N Doshi M Naumova EN Assessing seasonal variation in multisource surveillance data: annual harmonic regression Intelligence and Security Informatics: Biosurveillance 2007 Berlin Heidelberg: Springer 114 123

[B21] Estrada-Peña A Estrada-Sánchez A Estrada-Sánchez D de la Fuente J Assessing the effects of variables and background selection on the capture of the tick climate niche Int J Hlth Geog 2013 12 43 55 10.1186/1476-072X-12-43 PMC3849650 24069960

[B22] Reisen WK Landscape epidemiology of vector-borne diseases Ann Rev Entomol 2010 55 461 483 19737082 10.1146/annurev-ento-112408-085419

[B23] Pettorelli N Ryan S Mueller T Bunnefeld N Jedrzejewska B Lima M Kausrud K The normalized difference vegetation index (NDVI): unforeseen successes in animal ecology Climate Res 2011 46 15 27

[B24] Hamel S Garel M Festa-Bianchet M Gaillard JM Côté SD Spring Normalized Difference Vegetation Index (NDVI) predicts annual variation in timing of peak faecal crude protein in mountain ungulates J Appl Ecol 2009 46 582 589

[B25] Huete A Didan K Miura T Rodriguez EP Gao X Ferreira LG Overview of the radiometric and biophysical performance of the MODIS vegetation indices Remote Sens Environ 2002 83 195 213

[B26] Myneni RB Hoffman S Knyazikhin Y Privette JL Glassy J Tian Y Running SW Global products of vegetation leaf area and fraction absorbed PAR from year one of MODIS data Remote Sens Environ 2002 83 214 231

[B27] Chen J Jönsson P Tamura M Gu Z Matsushita B Eklundh L A simple method for reconstructing a high-quality NDVI time-series data set based on the Savitzky–Golay filter Remote Sens Environ 2004 91 332 344

[B28] Hmimina G Dufrêne E Pontailler JY Delpierre N Aubinet M Caquet B Soudani K Evaluation of the potential of MODIS satellite data to predict vegetation phenology in different biomes: An investigation using ground-based NDVI measurements Remote Sens Environ 2013 132 145 158

[B29] R Development Core Team: R A language and environment for statistical computing 2012 Vienna, Austria: R Foundation for Statistical Computing ISBN 3-900051-07-0, URL http://www.R-project.org/

[B30] Hijmans RJ van Etten J: R Geographic data analysis and modeling. R package version 2.0-41 2012 http://CRAN.R-project.org/package=raster

[B31] Kung-Sik C Ripley B TSA: Time Series Analysis. R package version 1.01 2012 http://CRAN.R-project.org/package=TSA

[B32] Cawsey EM Austin MP Baker BL Regional vegetation mapping in Australia: a case study in the practical use of statistical modelling Biodiversity Conserv 2002 11 2239 2274

[B33] Elith J Burgman MA Regan HM Mapping epistemic uncertainties and vague concepts in predictions of species distributions Ecol Modell 2002 157 313 329

[B34] Nakazawa M fmsb: Functions for medical statistics book with some demographic data. R package version 0.4.3 2013 http://CRAN.R-project.org/package=fmsb

[B35] Hijmans RJ Cameron SE Parra JL Jones PG Jarvis A Very high resolution interpolated climate surfaces for global land areas Int J Climatol 2005 25 1965 1978

[B36] Estrada-Peña A Bouattour A Camicas JL Guglielmone A Horak I Jongejan F Walker AR The known distribution and ecological preferences of the tick subgenus *Boophilus* (Acari: Ixodidae) in Africa and Latin America Exp Appl Acarol 2006 38 219 235 16596355 10.1007/s10493-006-0003-5

[B37] Rogers DJ Randolph SE The global spread of malaria in a future, warmer world Science 2000 289 1763 1766 10976072 10.1126/science.289.5485.1763

[B38] Rogers DJ Randolph SE Snow RW Hay SI Satellite imagery in the study and forecast of malaria Nature 2002 415 710 715 11832960 10.1038/415710a PMC3160466

[B39] Delong ER Delong DM Clarke-Pearson DL Comparing the areas under two or more correlated receiver operating characteristic curves: a nonparametric approach Biometrics 1988 44 837 845 3203132

[B40] Elith J Graham CH Anderson RP Dudik M Ferrier S Guisan A Hijmans RJ Huettmann F Leathwick JR Lehmann A Li J Lohmann LG Loiselle BA Manion G Moritz C Nakamura M Nakazawa Y Overton J Peterson AT Phillips SJ Richardson KS Scachetti-Pereira R Schapire RE Soberón J Williams S Wisz MS Zimmermann NE Novel methods improve prediction of species’ distributions from occurrence data Ecography 2006 29 129 151

[B41] Kabthimer GT Assessment of spatio-temporal patterns of NDVI in response to precipitation using NOAA-AVHRR rainfall estimate and NDVI data from 1996–2008, Ethiopia Master’s Thesis 2012 Stockholm, Sweden: University of Stockholm

[B42] Caminade C Medlock JM Ducheyne E McIntyre KM Leach S Baylis M Morse AP Suitability of European climate for the Asian tiger mosquito *Aedes albopictus*: recent trends and future scenarios J Royal Soc Interface 2012 9 2708 2717 10.1098/rsif.2012.0138 PMC3427500 22535696

[B43] Haeberlein S Fischer D Thomas SM Schleicher U Beierkuhnlein C First assessment for the presence of phlebotomine vectors in Bavaria, Southern Germany, by combined distribution modeling and field surveys PLoS One 2013 8 e81088 24260539 10.1371/journal.pone.0081088 PMC3832422

[B44] Fischer D Moeller P Thomas SM Naucke TJ Beierkuhnlein C Combining climatic projections and dispersal ability: a method for estimating the responses of sandfly vector species to climate change PLoS Negl Trop 2011 5 e1407 10.1371/journal.pntd.0001407 PMC3226457 22140590

[B45] Pickles RS Thornton D Feldman R Marques A Murray DL Predicting shifts in parasite distribution with climate change: a multitrophic level approach Global Change Biol 2013 19 2645 2654 10.1111/gcb.12255 23666800

[B46] De Clercq EM Estrada-Peña A Adehan S Madder M Vanwambeke SO An update on distribution models for Rhipicephalus microplus in West Africa Geospatial Hlth 2013 8 301 308 10.4081/gh.2013.75 24258904

[B47] Soberón J Peterson AT Interpretation of models of fundamental ecological niches and species’ distributional areas Biodivers Inform 2005 2 1 10

[B48] Jiménez-Valverde A Acevedo P Barbosa AM Lobo JM Real R Discrimination capacity in species distribution models depends on the representativeness of the environmental domain Global Ecol Biogeog 2013 22 508 516

[B49] Merow C Smith MJ Silander JA A practical guide to MaxEnt for modeling species’ distributions: what it does, and why inputs and settings matter Ecography 2013 36 1058 1069

[B50] Guglielmone AA Robbins RG Apanaskevich DA Petney TN Estrada-Peña A Horak IG The hard ticks of the World (Acari: Ixodida: Ixodidae) 2014 New York: Springer 738

[B51] Madder M Adehan S De Deken R Adehan R Lokossou R New foci of *Rhipicephalus microplus* in West Africa Exp Appl Acarol 2012 56 385 390 22286115 10.1007/s10493-012-9522-4

[B52] Porretta D Mastrantonio V Mona S Epis S Montagna M Sassera D Urbanelli S The integration of multiple independent data reveals an unusual response to Pleistocene climatic changes in the hard tick Ixodes ricinus Mol Ecol 2013 22 1666 1682 23398505 10.1111/mec.12203

[B53] Medley KA Niche shifts during the global invasion of the Asian tiger mosquito, *Aedes albopictus* Skuse (Culicidae), revealed by reciprocal distribution models Global Ecol Biogeog 2010 19 122 133

[B54] Moffett A Shackelford N Sarkar S Malaria in Africa: vector species' niche models and relative risk maps PLoS One 2007 2 e824 17786196 10.1371/journal.pone.0000824 PMC1950570

[B55] Hay SI Tatem AJ Graham AJ Goetz SJ Rogers DJ Global environmental data for mapping infectious disease distribution Adv Parasitol 2006 62 37 77 16647967 10.1016/S0065-308X(05)62002-7 PMC3154638

[B56] Wheeler DC Diagnostic tools and a remedial method for collinearity in geographically weighted regression Environ Plan 2007 39 2464 2481

[B57] Synes NW Osborne PE Choice of predictor variables as a source of uncertainty in continental‒scale species distribution modelling under climate change Global Ecol Biogeog 2011 20 904 914

[B58] Bedia J Herrera S Gutierrez JM Dangers of using global bioclimatic datasets for ecological niche modeling. Limitations for future climate projections Global Planetary Change 2013 107 1 12

[B59] Eastman JR Fulk M Long sequence time series evaluation using standardized principal components Photogrammetric Eng Remote Sens 1993 59 991 996

[B60] Jönsson P Eklundh L TIMESAT-a program for analyzing time-series of satellite sensor data Comput Geosci 2004 30 833 845

