# Supplementary figures and images for: A global set of Fourier-transformed remotely sensed covariates for the description of abiotic niche in epidemiological studies of tick vector species
Source: Parasit Vectors. 2014 Jul 2;7:302. doi: 10.1186/1756-3305-7-302 (PMC4089935; doi:10.1186/1756-3305-7-302)

A

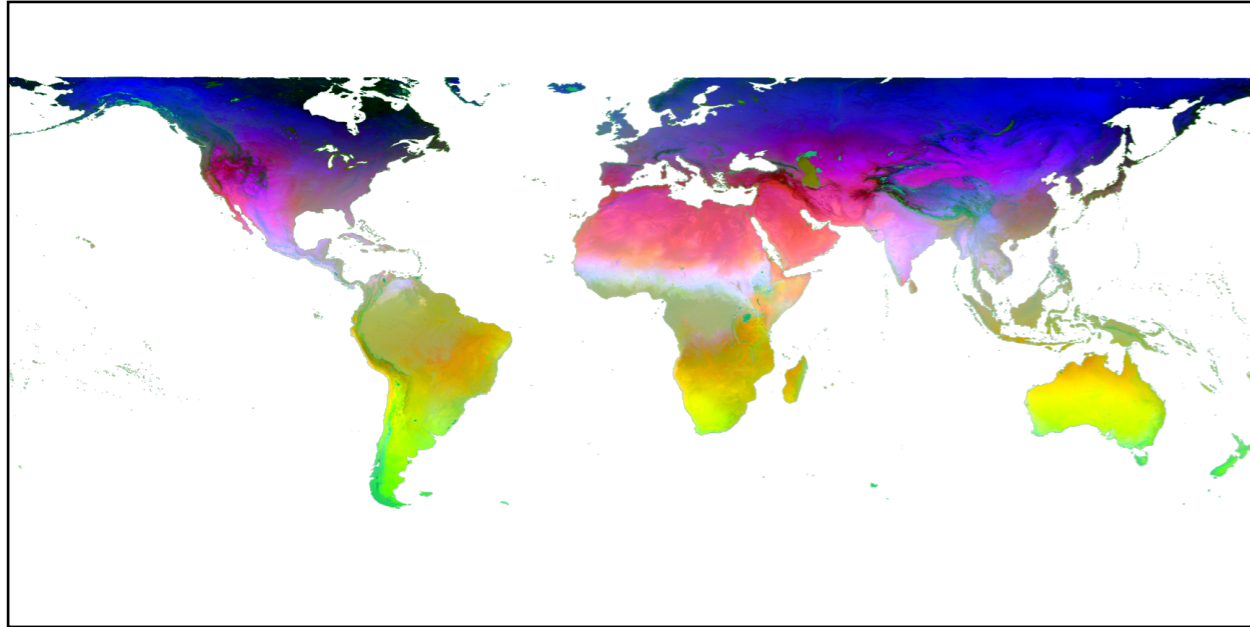

B

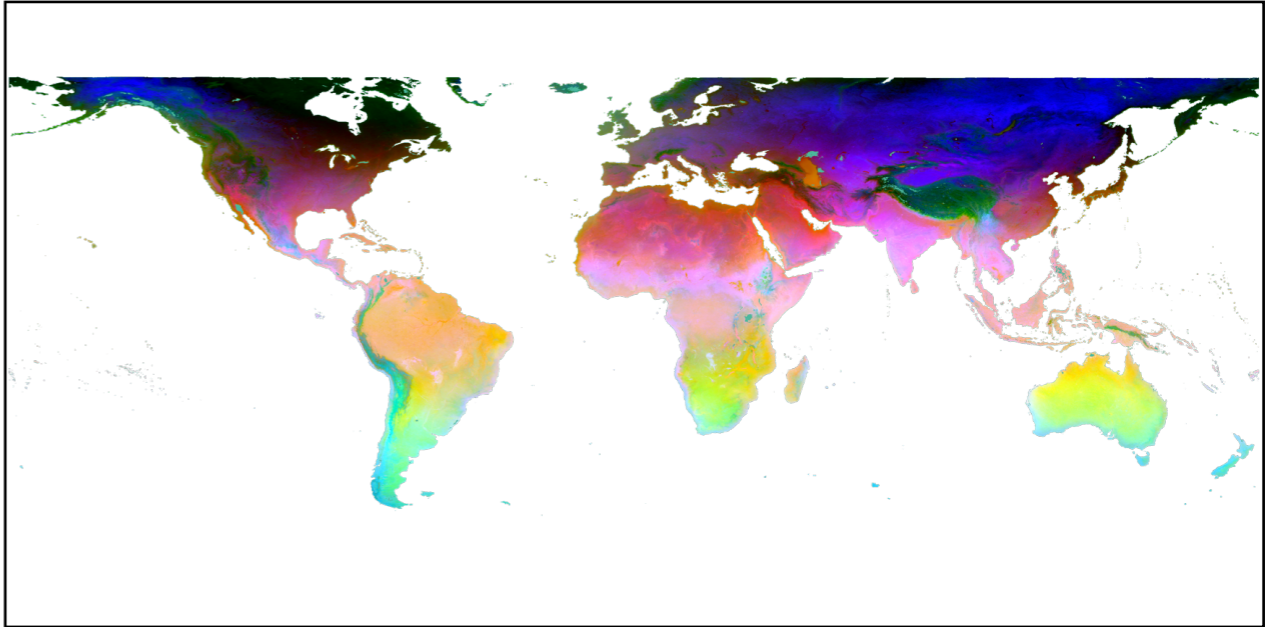

C

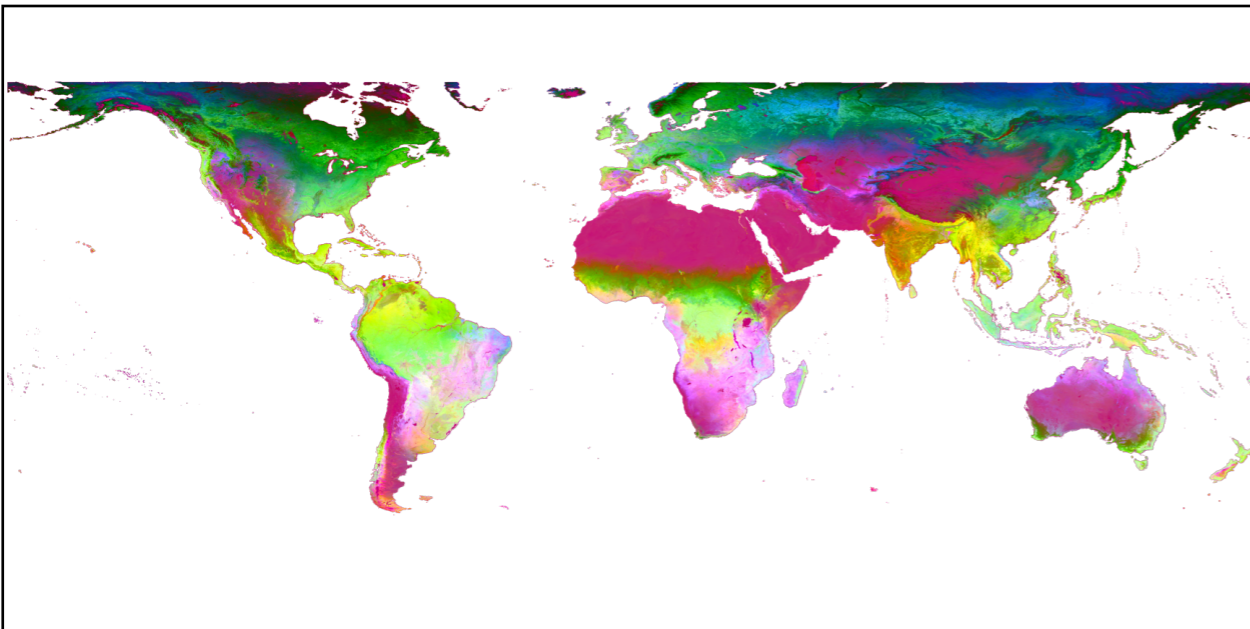

D

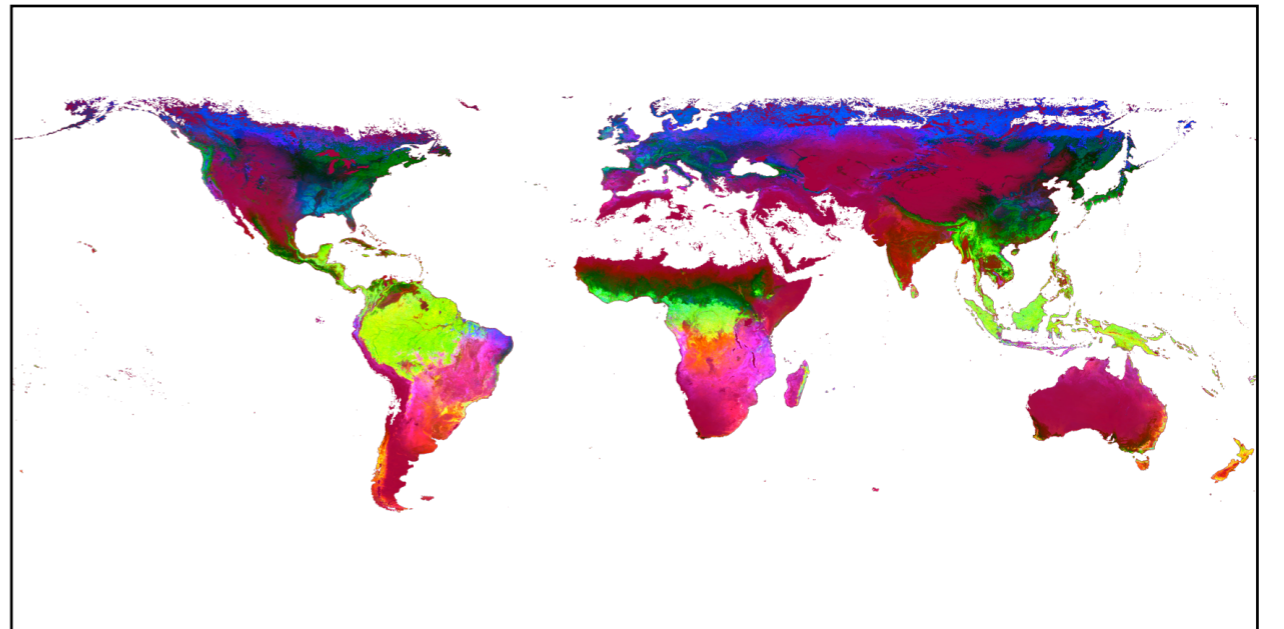

S1

Supplement: Additional file 2: Figure S1 — Composite images of the coefficients of harmonic regression for the four series of remotely sensed covariates. Composites represent LSTD (A), LSTN (B), NDVI (C), and LAI (D). Compositions related to LSTD and LSTN were prepared with A1 (red), A2 (blue), and A3 (green) coefficients (i.e., the three first coefficients of the harmonic regression for each variable). Compositions regarding NDVI and LAI were prepared with the A1 (green), A2 (blue), and A3 (red) coefficients. [file 1756-3305-7-302-S2.pdf]
